# Progress of Veterinary Science

**Published:** 1882-07

**Authors:** 


### PROGRESS OF VETERINARY SCIENCE.

---

Typhoid Fever in a Horse.—A case which was diagnosed as typhoid fever in a horse is reported by Dr. Ubele, of Künzelsan. The symptoms were severe fever, 103° to 107°; pulse, 60 to 110; suffused eyes; no appetite. On the ninth day, death occurred. Post mortem showed extensive exchymoses of the mucous membranes, and intense congestion about Peyer's patches.

An Ossifying Spinal Pachymeningitis in a Dog.—A four-year-old dog suffered for two weeks, apparently from an influenza. It grew weaker daily. Posterior paralysis and, finally, complete paralysis of the legs developed. The animal then died. Post mortem showed wide-spread catarrhs and broncho-pneumonia. On opening the spinal cord, evidences of a diffuse myelitis were found, while in the thickened dura mater were numerous bony plates one to one and a-half inches long.

Hair-balls in a Ten-days-old Calf.—Dr. B. Th. Leitner reports (*Zeitschrift f. Thiermedicin*) the case of a ten-days-old calf in whose œsophagus was found a hair-ball the size of an apple. It was probably formed during intra-uterine life by the calf swallowing the fluid in which it floated.

Dilated and Calcified Arteries in a Parrot Sixty-four Years Old.—At the post mortem held over the remains of a pet parrot (*Zeitschrift f. Thiermedicin*) it was seen that all the large arteries leading from the heart were lined with a whitish layer, and looked as though they had been injected with gypsum. This calcification was found to extend even to the small arteries of the legs, wings, and mesentery. The left ventricle of the heart was hypertrophied, the right dilated and thin.

The other organs showed that the animal had died from chronic inanition, due to deficient blood supply from the calcified arteries. There was a general venous conjestion with bleeding into stomach and intestines, and finally death from carbonic acid poisoning and heart failure.

Death from Asphyxia in a Swan.—Professor Bonnet describes the post mortem upon a swan which had died suddenly after feeding. The larynx was found completely filled with a piece of cheese. The swan did not consequently sing before he died.

Gall-stone in a Horse.—Dr. Gl. Marggraff reports a case of a horse which suffered for some time from jaundice. On post-mortem, a brownish-yellow gall-stone was found impacted in the bile duct. The liver showed evidence of congestion and interstitial inflammation, and was stained with pigments.

In answer to numerous inquiries regarding the health of Dr. C. D. House, we are glad to state that he is now convalescent, and is staying for the Summer in Connecticut.

Syringe for Veterinary Use.—Drs. Findeisen and Vogel, German veterinarians, have extensively used an injection syringe made on the principle of the Davidson syringe, but much larger. Vogel has found it of great value in uterine catarrhs, fluor albus, sterility, retained placenta, as also in colic and constipation.

Kidney Disease in the Cow is, according to Leimer (*Repertorium der Thierheilkunde*) most frequently of the form known as *interstitial nephritis.* A parenchymatous change is often associated with this.

Bile Pigments in the Urine of the Dog. — Dr. Eugen Fröhner (*Deutches Zeitschrift fur Thiermedicin*) asserts that, normally, there are none of the bile-coloring matters in the dog's urine. When these are found it indicates either (1) a gastro-intestinal catarrh or (2) a lowering of blood pressure in the internal organs, especially the liver. This latter may be produced by injuries and bleedings of external organs.

Experiments with Huch's Kraft-futter or Blood-meal on Horses. —This special food for horses contains a large amount of albuminous substances (36 per cent.), considerable fat (1.2 per cent.), phosphates and other salts. It has been tried upon army horses by Dr. Findeisen of Ulm. The regular daily ration for each horse was: oats, 10½ lbs.; hay, 5 lbs.; straw, 7 lbs. To this was added 1½ of the "blood-meal." The horses eat it with great avidity. The four horses experimented upon gained an average of 20 lbs. in a month, and improved very much in general condition.

Epidemic Nose-bleeding and Pernicious Anæmia in Dogs. — M. Meguin reports that in the disease called epidemic bleeding of the nose of dogs, there is a chronic inflammation of the mucous membrane of the small intestine and cœcum, in which the membrane and villosities are thickened and reddened. These lesions alone would explain the anæmia, but they are produced by the bites of numerous worms (*ankylostonum* and *tricho-cephalus.*) The same condition has been found to exist in cats.

Remedy for Lice.—Lice on cattle may be easily and thoroughly destroyed by a tar ointment. Melt together an ounce of tar and a pound of lard, and stir while cooling. Apply a little on the parts most affected. It may even be applied lightly over the whole skin without danger to the animal. The same good results may be obtained from boiling an ounce of tobacco in a quart of water and sponging the parts with the solution.—*National Live Stock Journal.*

Goitre in the Lower Animals.—Goitre is not an affection peculiar to man alone. M. Adam, veterinary surgeon at Augsburg, has remarked that after staying a certain length of time in that city a number of horses were attacked with goitre. There are only a certain number of stables where goitre shows itself, all situated at the east of the city. It is impossible to determine the cause of it. Goitre is not rare with the dogs of Augsburg, and is unusually frequent with the inhabitants of that city. The disease does not prevail in Switzerland, however.—*Medical Record.*

Diphtheria in Calves Communicated to Pigs.—Mr. Cole, a veteritarry surgeon of Hinckley, in Australia, has published the following illusnation of the way in which diphtheria may be communicated from one of the domestic animals to another of a different species, thus indicating special sources from which the human disease may at times be contracted. A calf, about five months old, was found to be dying with some symptoms of a throat disorder, and instructions were given to have the body buried, which through some neglect was not done immediately, so that a sow which managed to get access to the enclosure, attacked the diseased meat and ate some of it. This circumstance came to be known when, a few days later, some of the pigs were taken down with throat disease. Eventually the sow and her young pigs were also victims. These latter died within twenty-four hours, while the others, including a boar, recovered entirely. Apropos of this outbreak among domestic animals an account is given of an epidemic that occurred in the Oakleigh police station, the disease being on this occasion traced to a diseased cow, whose milk had been used by the inmates of the station.—*Australian Veterinary Journal.*

Effect of Arsenic on Cats.—Chronic poisoning by arsenic has received the experimental attention of Drs. Caillot de Poncy and Livon, and the results of their observations may be of value to certain ladies and not a few medical practitioners. Small doses were given to cats at intervals. Under the influence of arsenic they were able to take more than the normal quantity of food. For a time they increased in weight, and presented every outward evidence of good health. By and by a change occurred. The cats had diarrhœa; they lost appetite; they became languid, and they died in an anæmic and lean condition.

Human and Bovine Tuberculosis.—In the *Italia Medica*, the paper read by Dr. Bizzozzero on the above subject, at the International Medical Congress, held at Turin, is published, and in it are held the following conclusions: Human and bovine tuberculosis have between them the closest anatomical affinity. When the matters of both are inoculated in susceptible animals, they equally give rise to the development of tubercular neoformations—a result which is not produced by inoculation with other substances. It must, therefore, be admitted that their virus is identical; and this shows the necessity for resorting to severe prophylactic measures in order to prevent the transmission of the disease from animals to man.—*National Live Stock Journal.*

Resuscitation of Animals after Exposure to Extreme Cold.—F.F. Loptschinski says (*Vratsch*, No. 5–7) there is a remarkable disagreement between experimenters and clinical observers as to the manner of treating individuals that have been exposed to extreme cold. While nearly all of the latter hold that the application of heat should be gradual, the former (Beck, Horwat, Jacoby) advise that it should be rapid. The author has experimented with dogs to solve this question. Some of the animals were exposed to cold air (—17° C., two above zero F.); others were packed in freezing mixtures (—13—15° C., 5—8° F.). After freezing, one of the animals (twenty experiments were made, each with three dogs) was immediately placed in a warm bath of 37° R. (115° F.); the second was placed in a room the temperature of which was 22—24° R. (81—86° F.); the third was placed in a temperature of 0° (32° F.), and then as the temperature of the rectum arose and manifestations of life were shown, the bodily temperature increased. Friction with brushes and dry cloths were used in all three cases. The experiments, which were made with great care, throw light on the various conditions, which will not be referred to here (blood examinations, microscopic examinations of the muscular tissue, conditions of temperature, etc.); but there were other results which have a practical significance for physicians. Of twenty animals that were exposed to a low temperature, which was gradually elevated, fourteen died; of twenty animals that were immediately brought into a warmer department, eight died; of twenty animals that were immediately placed in a warm bath, *none* died.

